# Supplementary material for: Superexchange-stabilized long-distance Cu sites in rock-salt-ordered double perovskite oxides for CO2 electromethanation
Source: Nat Commun. 2024 Feb 21;15:1565. doi: 10.1038/s41467-024-45747-5 (PMC10879110; doi:10.1038/s41467-024-45747-5)
Supplement: Supplementary file 1 — Supplementary Information [file 41467_2024_45747_MOESM1_ESM.pdf]

Supplementary Information

Superexchange-stabilized long-distance Cu sites in rock-salt-ordered double perovskite oxides for CO_2 electromethanation

Jiawei Zhu[1,2,3,10,*], Yu Zhang[1,2,3,4,10], Zitao Chen[5], Zhenbao Zhang[6], Xuezeng Tian[5], Minghua Huang[7], Xuedong Bai[5], Xue Wang[8], Yongfa Zhu[9], Heqing Jiang[1,2,3,*]

[1]Qingdao Institute of Bioenergy and Bioprocess Technology, Chinese Academy of Sciences, Qingdao 266101, China

[2]Shandong Energy Institute, Qingdao 266101, China

[3]Qingdao New Energy Shandong Laboratory, Qingdao 266101, China

[4]University of Chinese Academy of Sciences, Beijing 100049, China

[5]Beijing National Laboratory for Condensed Matter Physics and Institute of Physics, Chinese Academy of Sciences, Beijing 100190, China

[6]School of Chemistry and Chemical Engineering, Linyi University, Linyi 276005, China

[7]School of Materials Science and Engineering, Ocean University of China, Qingdao 266100, China

[8]School of Energy and Environment, City University of Hong Kong, Hong Kong 999077, China

[9]Department of Chemistry, Tsinghua University, Beijing 100084, China

[10]Jiawei Zhu and Yu Zhang contributed equally to this work

*Email: zhujw@qibebt.ac.cn, jianghq@qibebt.ac.cn

Supplementary Figures

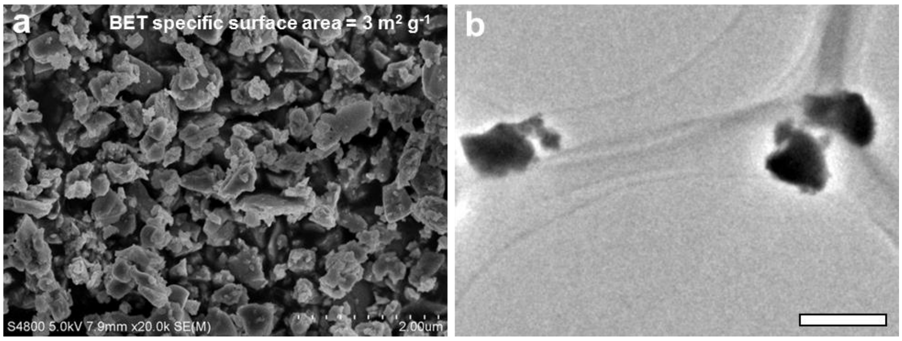

Supplementary Fig. 1. (a) SEM image of Sr_2CuWO_6. (b) TEM image of Sr_2CuWO_6 (scale bar: 500 nm). (BET: Brunauer-Emmett-Teller)

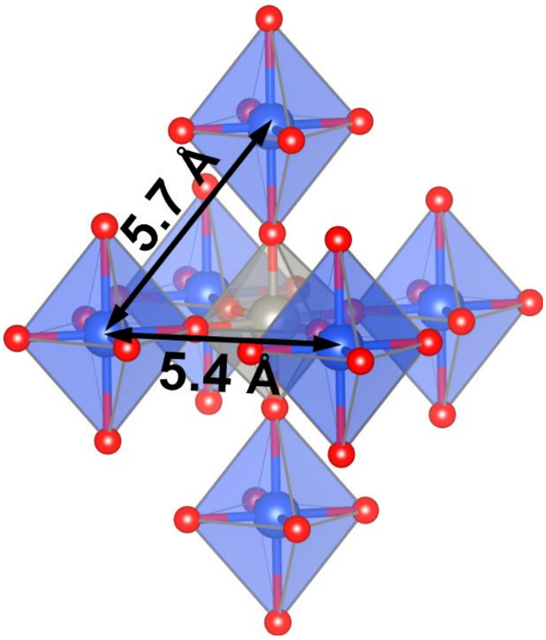

Supplementary Fig. 2. Schematic illustrations of the distances between the probably nearest Cu cations in the tetragonal *I*4/*m* phase. Cu, W, and O are represented by blue, gray, and red dots, respectively. The blue and gray octahedra represent CuO_6 and WO_6 motifs, respectively.

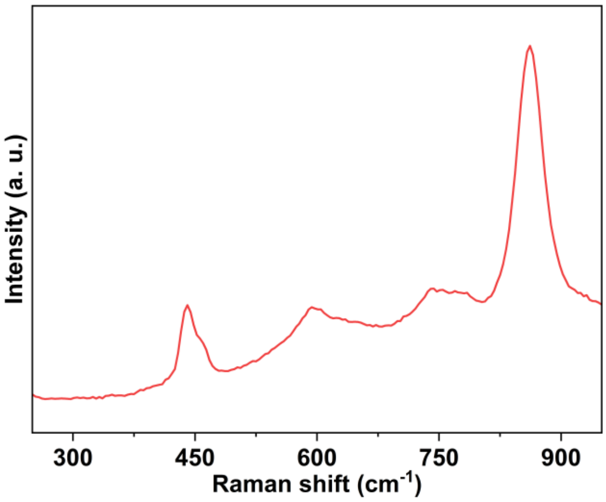

Supplementary Fig. 3. Raman spectra of Sr_2CuWO_6.

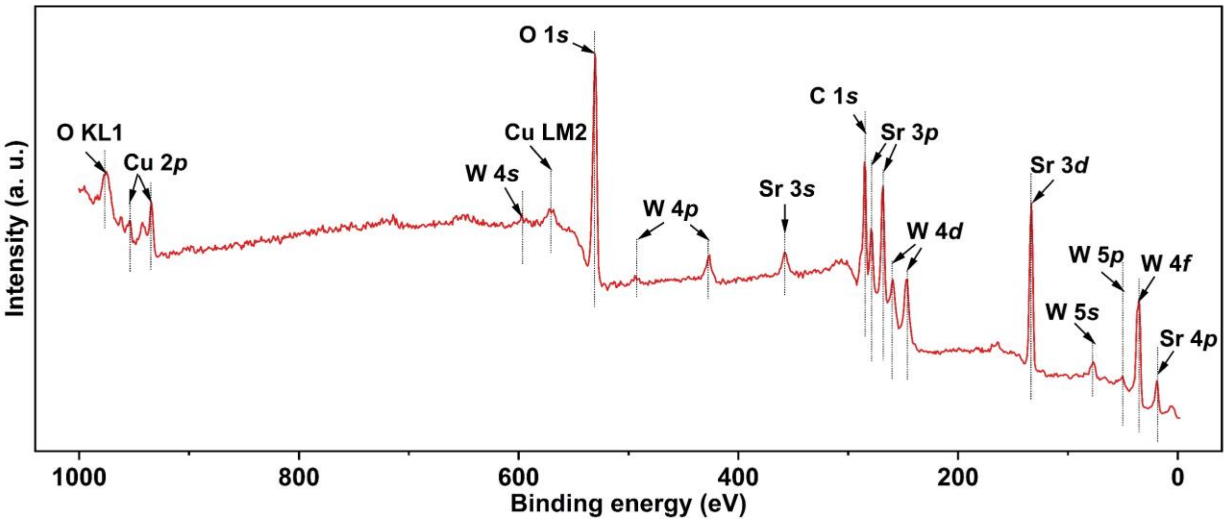

Supplementary Fig. 4. Wide-scan XPS spectra of Sr_2CuWO_6.

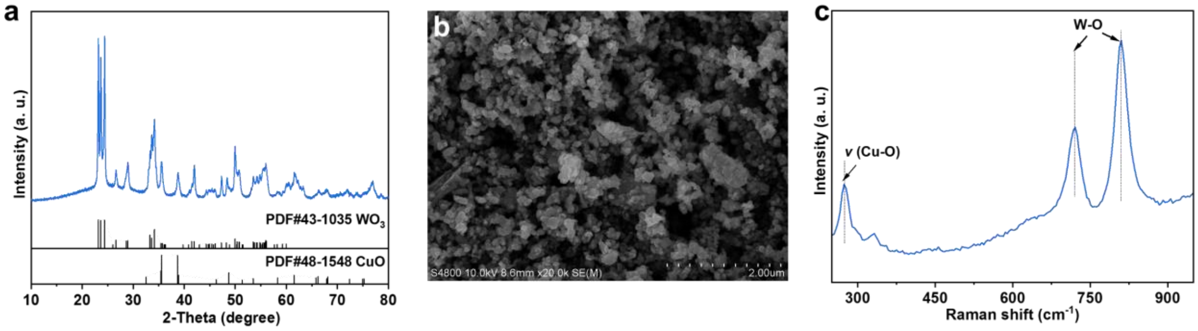

Supplementary Fig. 5. (a) XRD pattern, (b) SEM image, and (c) Raman spectra of CuO/WO_3.

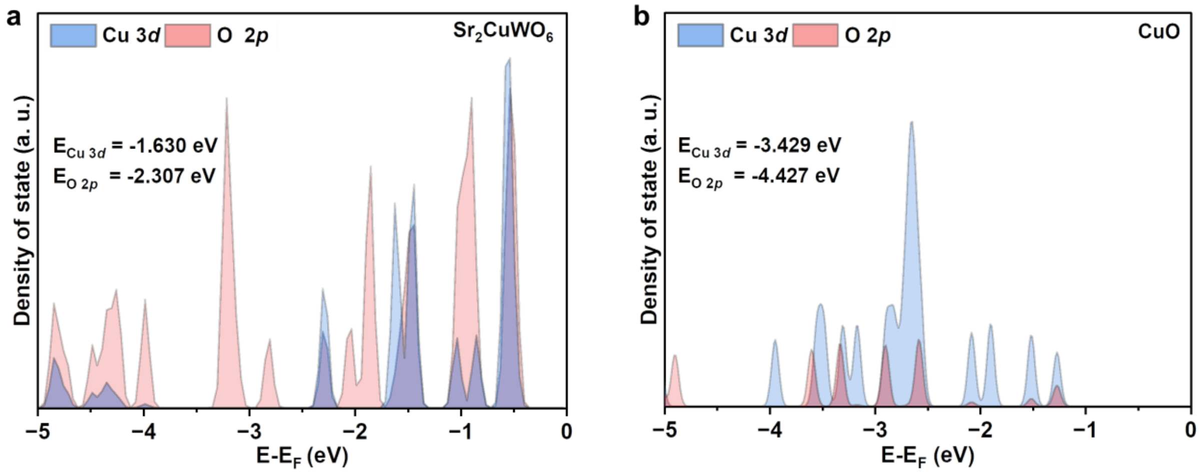

Supplementary Fig. 6. DFT-calculated projected density of states (PDOS) of Cu 3*d* and O 2*p* orbitals in (a) Sr_2CuWO_6 and (b) CuO.

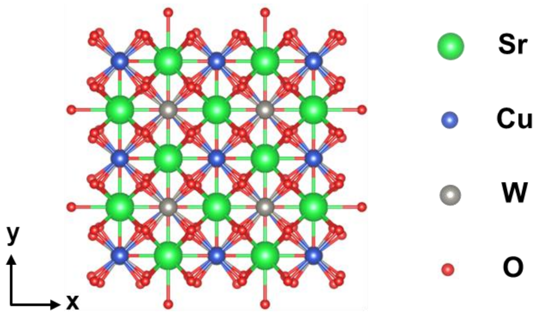

Supplementary Fig. 7. Top view of the CuO_2/WO_2-terminated Sr_2CuWO_6(001) surface.

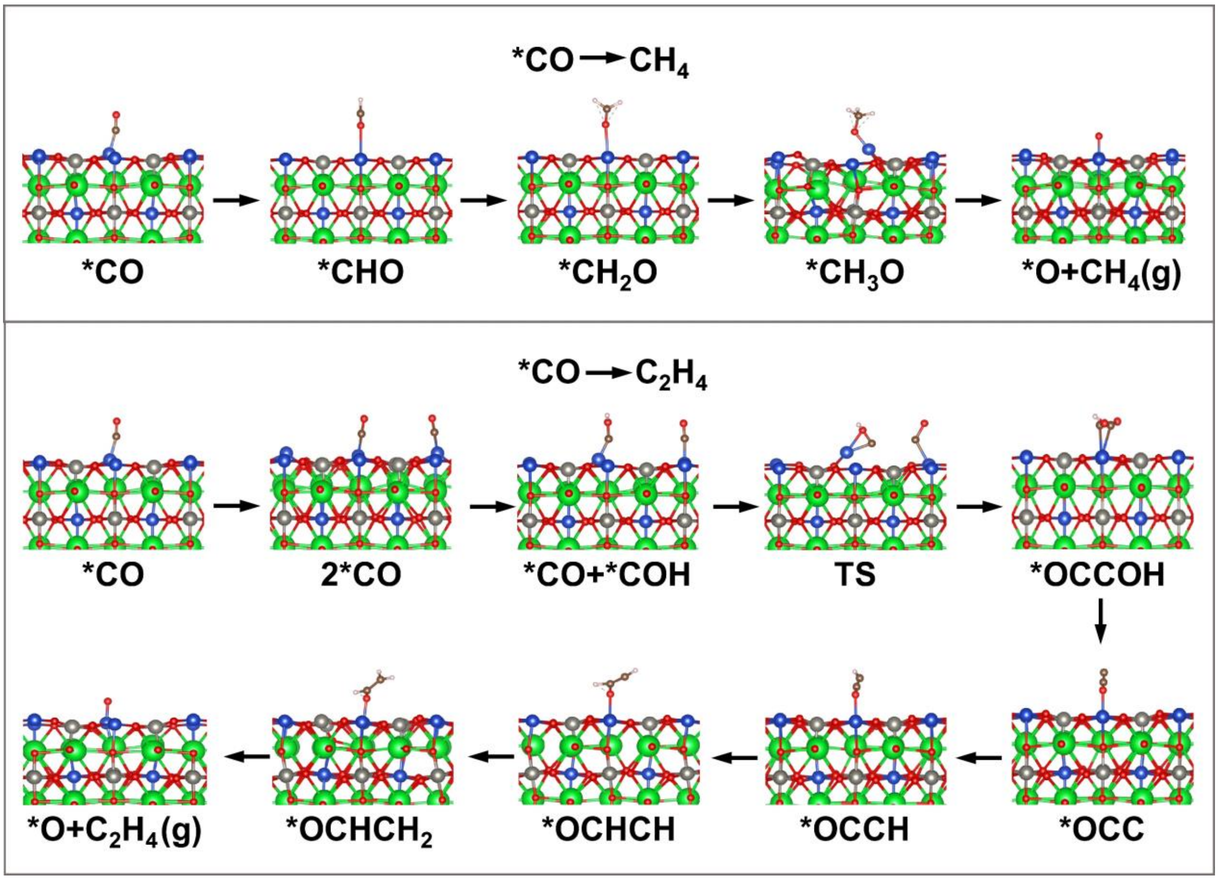

Supplementary Fig. 8. DFT-calculated structural details of intermediates on the Sr_2CuWO_6(001) surface. Cu, W, O, C, and H are represented by blue, gray, red, brown, and white dots, respectively.

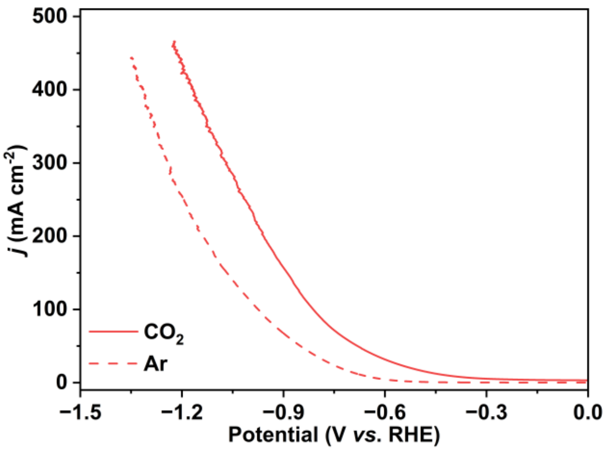

Supplementary Fig. 9. LSV curves of the Sr_2CuWO_6 catalyst measured in CO_2- or Ar-flowed liquid-electrolyte flow cell.

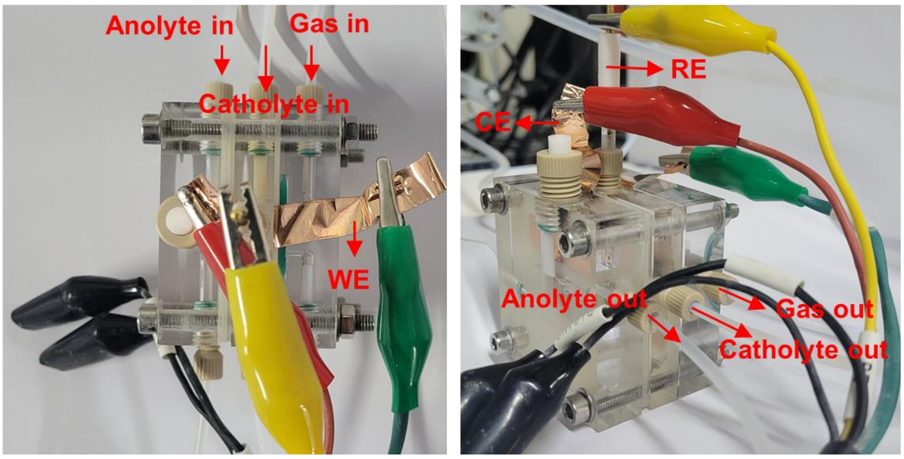

Supplementary Fig. 10. The images of the flow cell for CO_2RR measurement.

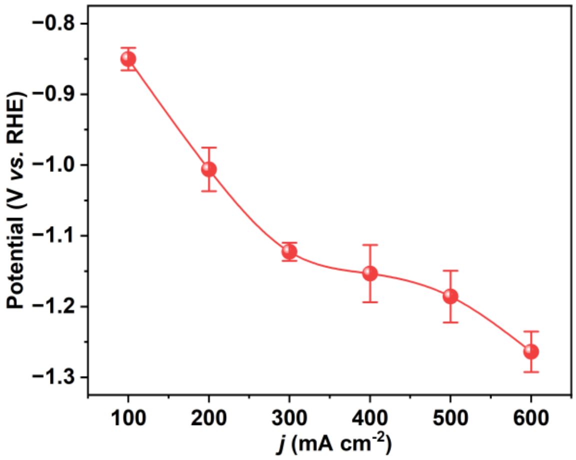

Supplementary Fig. 11. The potentials of Sr_2CuWO_6 at corresponding applied current densities. The error bars represent the mean ± SD (n = 3 replicates).

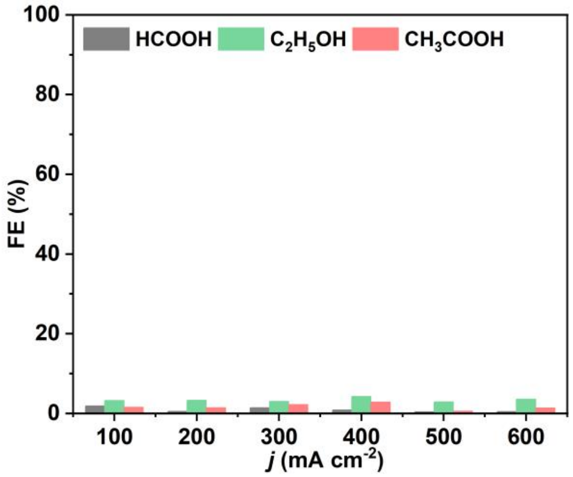

Supplementary Fig. 12. FEs for various liquid products over the Sr_2CuWO_6 catalyst at different applied current densities from a single measurement.

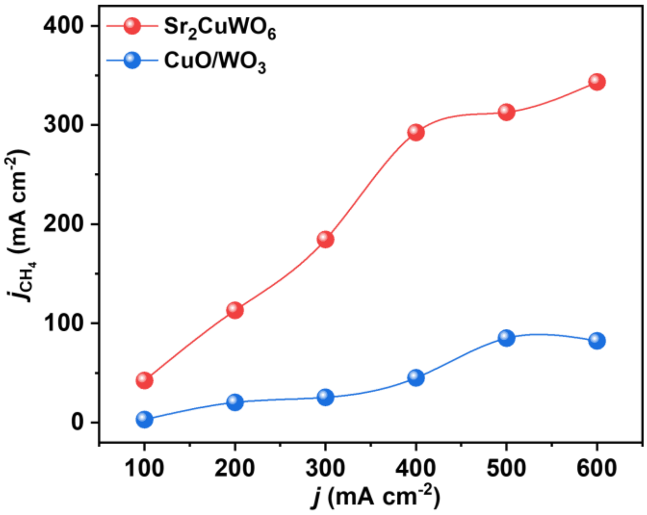

Supplementary Fig. 13. j_{CH_4} of the Sr_2CuWO_6 and CuO/WO_3 catalysts at different applied current densities (an average value from three independent experiments).

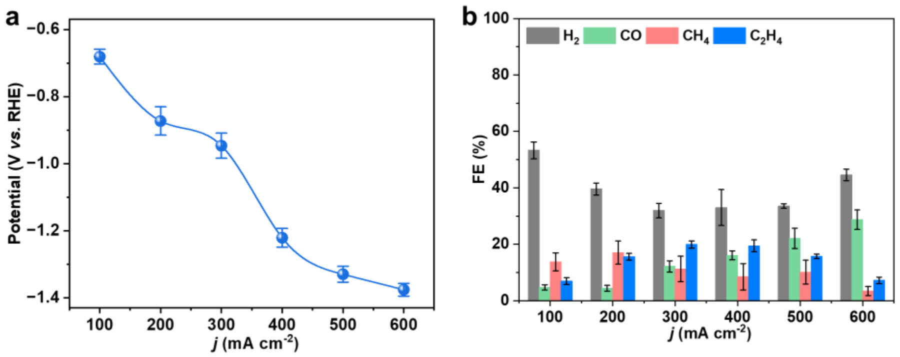

Supplementary Fig. 14. (a) The potentials of the CuO/WO_3 catalyst at corresponding applied current densities. (b) FEs for various CO_2RR products over the CuO/WO_3 catalyst. The error bars represent the mean ± SD (n = 3 replicates).

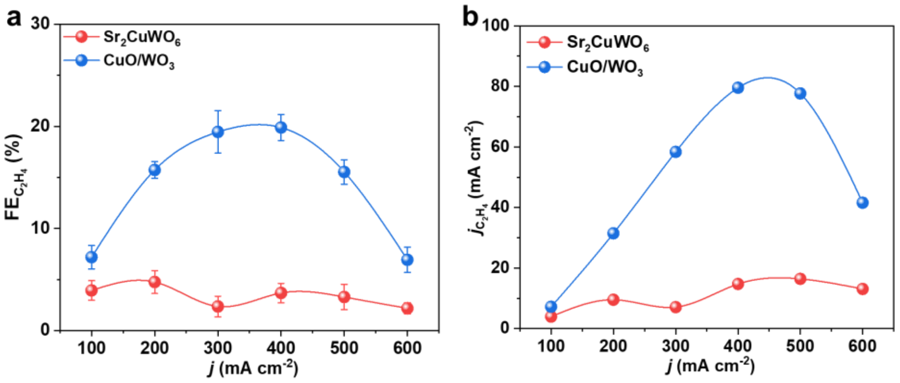

Supplementary Fig. 15. (a) $FE_{C_2H_4}$ and (b) $j_{C_2H_4}$ of the Sr_2CuWO_6 and CuO/WO_3 catalysts at different applied current densities. The error bars represent the mean ± SD (n = 3 replicates). $j_{C_2H_4}$ was an average value calculated from the $FE_{C_2H_4}$ and the applied current density.

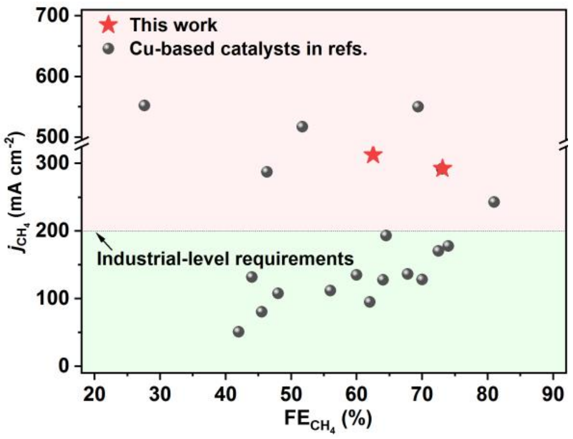

Supplementary Fig. 16. FE_{CH_4} and j_{CH_4} of the Sr_2CuWO_6 in comparison with those of most reported representative Cu-based catalysts in flow cells (Supplementary Table 5).

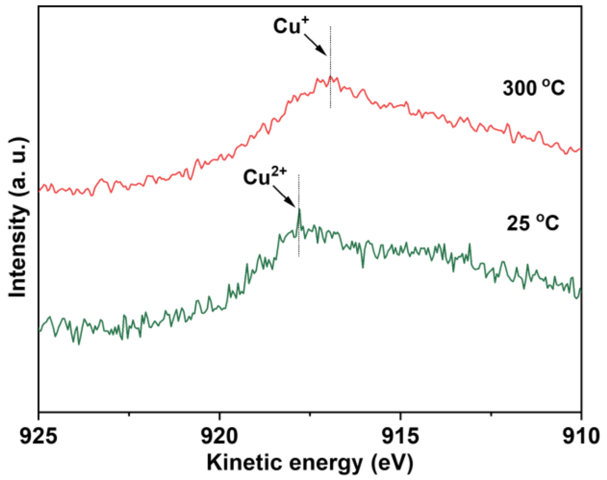

Supplementary Fig. 17. Cu LMM XPS spectra of Sr_2CuWO_6 after high-temperature reducing-atmosphere treatment.

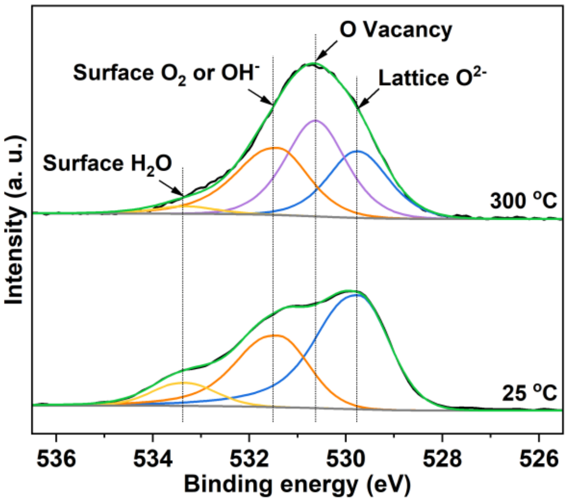

Supplementary Fig. 18. O 1*s* XPS spectra of Sr_2CuWO_6 after high-temperature reducing-atmosphere treatment.

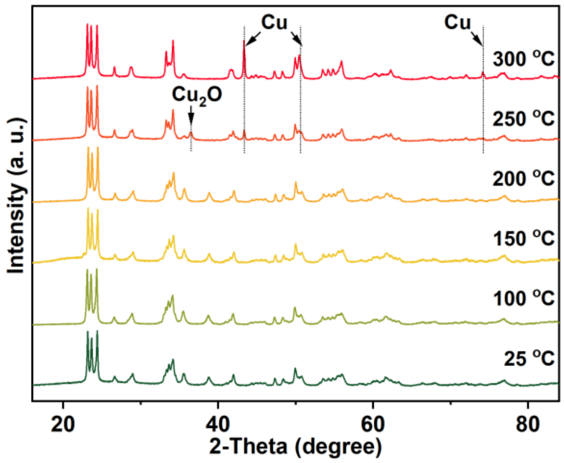

Supplementary Fig. 19. XRD patterns of the CuO/WO_3 catalyst after high-temperature reducing-atmosphere treatment.

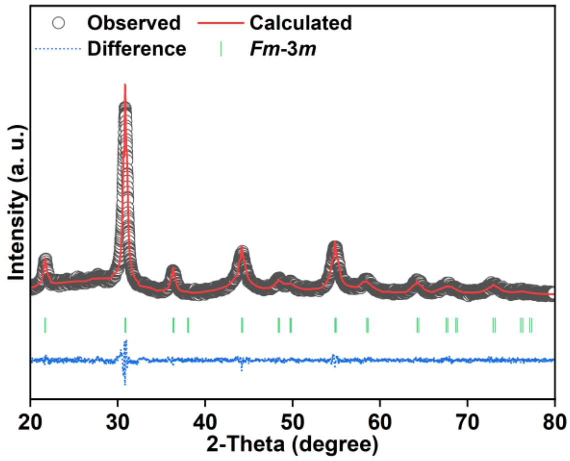

Supplementary Fig. 20. *Rietveld* refinement plot of XRD data from Sr_2CuWO_6 after 300 ºC reducing-atmosphere treatment.

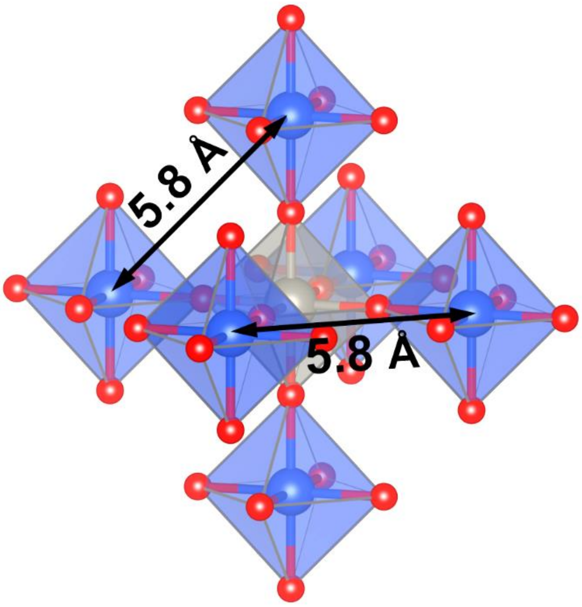

Supplementary Fig. 21. Schematic illustrations of the distances between the probably nearest Cu cations in the cubic *Fm*-3*m* phase. Cu, W, and O are represented by blue, gray, and red dots, respectively. The blue and gray octahedra represent CuO_6 and WO_6 motifs, respectively.

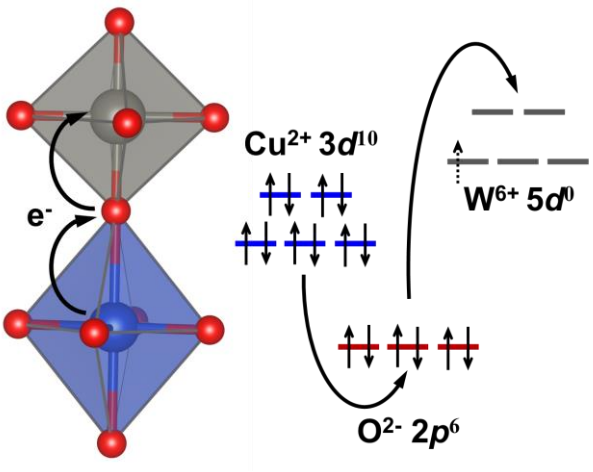

Supplementary Fig. 22. Schematic illustrations of Cu^{+}-O^{2-}-W^{6+} superexchange interaction. Cu, W, and O are represented by blue, gray, and red dots, respectively. The blue and gray octahedra represent CuO_6 and WO_6 motifs, respectively.

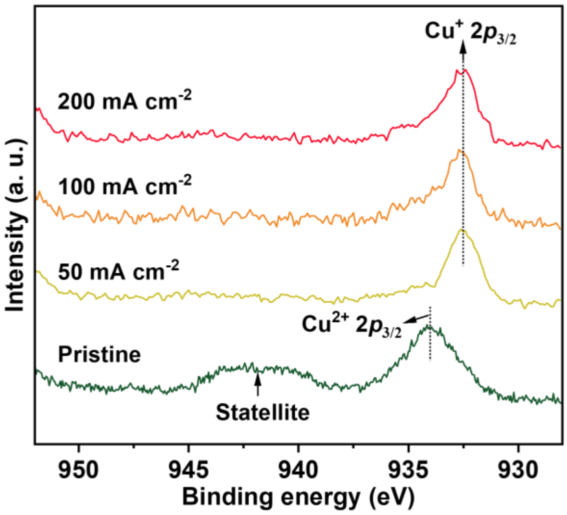

Supplementary Fig. 23. Cu 2*p* XPS spectra of the Sr_2CuWO_6 after CO_2RR at different current densities.

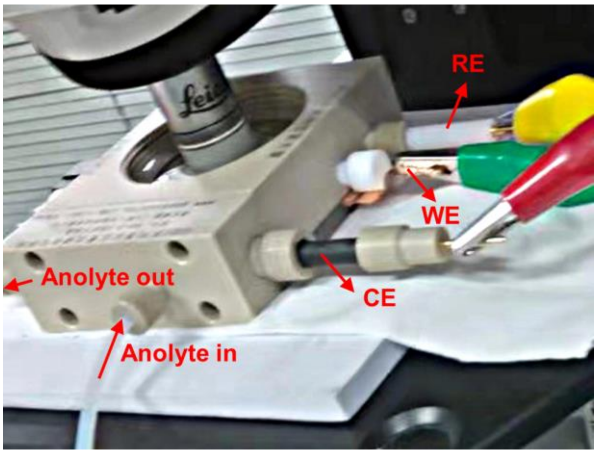

Supplementary Fig. 24. The image of the electrochemical operando electrolyzer (C031-2, Tianjin Gaoss Union Technology) for the in-situ Raman test.

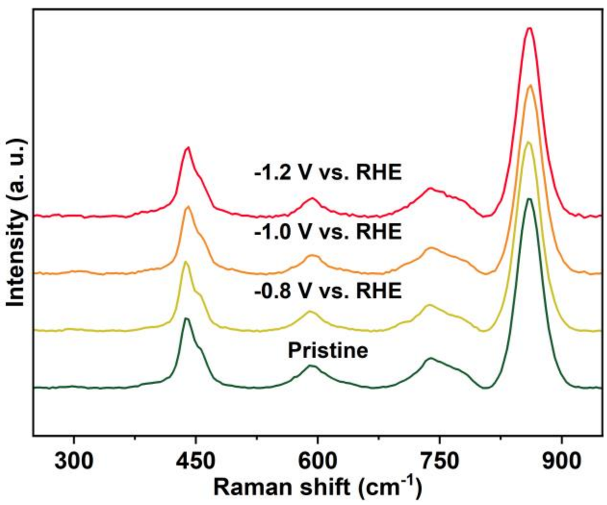

Supplementary Fig. 25. In-situ Raman Spectra of Sr_2CuWO_6 at different applied potentials.

Supplementary Tables

Supplementary Table 1. ICP-MS result of the Sr_2CuWO_6 catalyst.

Sample	Theoretical atomic ratio of metal elements	Tested atomic ratio of metal elements
Sr_2CuWO_6	Sr: Cu: W = 2: 1: 1	Sr: Cu: W = 2.01: 1: 1.01

Supplementary Table 2. Refined parameters of the Sr_2CuWO_6 catalyst from Rietveld refinement analysis using the corresponding XRD data.

Weight fraction	Space group	Lattice parameters	Reliability factors
100 wt%	*I4/m*	a = 5.43607 Å<br>b = 5.43607 Å<br>c = 8.40045 Å	R_{wp} = 5.77%<br>R_p = 3.70%<br>R_{exp} = 1.28%

Supplementary Table 3. Calculated average Bader charge |e| of surface Cu atoms and W atoms in the CuO, WO_3, and Sr_2CuWO_6.

Atom	CuO	WO_3	Sr_2CuWO_6	Variation
Cu	1.08 |e|	--	1.27 |e|	0.19 |e|
W	--	3.075 |e|	2.95 |e|	0.125 |e|

Supplementary Table 4. Comparison of the activity and selectivity for CH_4 from various Cu-based perovskite oxides.

Catalyst	Electrolyte	Potential (V *vs.* RHE)	FE_{CH_4}	j_{CH_4} (mA cm^{-2})	Ref.
La_2CuO_4 NBs	0.1 M $KHCO_3$	-0.9	31%	~1	1
La_2CuO_4 NRs	0.1 M $KHCO_3$	-0.9	30%	~0.2	1
bulk La_2CuO_4	0.1 M $KHCO_3$	-0.8	14%	~0.3	1
Cu/La_2CuO_4	0.1 M $KHCO_3$	-1.4	55%	116	2
$La_{1.7}CuO_4$	1 M KOH	-1.4	22%	25	3
$La_{1.8}CuO_4$	1 M KOH	-1.4	14%	17	3
$La_{1.9}CuO_4$	1 M KOH	-1.4	9%	12	3
La_2CuO_4	1 M KOH	-1.4	14%	18	3
$La_{1.8}Sr_{0.2}CuO_4$	0.5 M KOH	-1.74	14%	42	4
Cu_2O/La_2CuO_4	0.5 M $NaHCO_3$	-0.4	4%	~0.04	5
Sr_2CuWO_6	1 M KOH	-1.15	73.1%	292.4	This work
Sr_2CuWO_6	1 M KOH	-1.19	62.5%	312.8	This work

Supplementary Table 5. Comparison of the activity and selectivity for CH_4 from the most reported representative Cu-based catalysts in flow cell.

Catalyst	Electrolyte	Potential (V *vs.* RHE)	FE_{CH_4} (%)	j_{CH_4} (mA cm^{-2})	Ref.
Ca_2CuO_3	1 M KOH	-0.3	51.7%	517	6
$CuGaO_2$	1 M KOH	-1.7	69.4%	~550	7
$Li_{2-x}CuO_2$-10	1 M KOH	-3.3	27.6%	552	8
Ag@Cu_2O	1 M KOH	-1.2	74%	178	9
$CuSiO_x$	1 M KOH	-1.6	72.5%	170.4	10
Cu-Ce-O_x	1 M KOH	-0.82	67.8%	136.3	11
Cu_3N_x-50-µA	1 M KOH	N.A.	35.2%	352	12
Cu-porphyrin	0.5 M PBS	-1.6	70%	128.1	13
CoO/Cu/PTFE	1 M $KHCO_3$	-1.1	60%	135	14
Cu-FeSA	1 M $KHCO_3$	-1.1	64%	128	15
Cu SAs/GDY	1 M KOH	-1.2	81 %	243	16
Cu/p-Al_2O_3 SAC	1 M KOH	-1.2	62%	94.9	17
2% Cu-CeO_2 NR	1 M KOH	-0.9	45.5%	80.6	18
Cu/CeO_2	1 M KOH	-0.89	42%	51	19
Cu-PzI	1 M KOH	-1.0	46.3%	287.5	20
BNC-Cu	0.5 M $KHCO_3$	-1.46	73%	292	21
Cu octahedra	1 M KOH	-0.96	44%	132	22
sputter Cu	1 M $KHCO_3$	-1.42	48%	108	23
La_5Cu_{95}	1 M KOH	-1.72	64.5%	193.5	24
Au-Cu	1 M $KHCO_3$	-1.24	56%	112	25
Sr_2CuWO_6	1 M KOH	-1.15	73.1%	292.4	This work
Sr_2CuWO_6	1 M KOH	-1.19	62.5%	312.8	This work

Supplementary Table 6. O 1*s* XPS peak fitting parameters for Sr_2CuWO_6 after 300 °C reducing-atmosphere treatment.

Pristine

Name	Peak position (eV)	FWHM	Peak area
Surface H_2O	533.32	1.54	5807.94
Surface O_2 or OH^-	531.40	1.54	21967.29
Lattice O^{2-}	529.74	1.54	36039.39

300 °C

Name	Peak position (eV)	FWHM	Peak area
Surface H_2O	533.32	1.54	1873.54
Surface O_2 or OH^-	531.40	1.54	20667.73
O Vacancy	530.62	1.54	24095.95
Lattice O^{2-}	529.74	1.54	16790.48

Supplementary Table 7. Refined parameters of Sr_2CuWO_6 after 300 °C reducing-atmosphere treatment from Rietveld refinement analysis using the corresponding XRD data.

Weight fraction	Space group	Lattice parameters	Reliability factors
100 wt%	*Fm*-3*m*	a = 8.19699 Å<br>b = 8.19699 Å<br>c = 8.19699 Å	R_{wp} = 3.51%<br>R_p = 4.47%<br>R_{exp} = 1.22%

Supplementary Table 8. W 4*f* XPS peak fitting parameters for Sr_2CuWO_6 after CO_2RR at different current densities

Pristine

Name	Peak position (eV)	FWHM	Peak area
W^{6+} $4f_{5/2}$	37.27	1.1	15780.15
W^{6+} $4f_{7/2}$	35.13	1.1	21046.31

50 mA cm^{-2}

Name	Peak position (eV)	FWHM	Peak area
W^{6+} $4f_{5/2}$	37.27	1.65	1041.67
W^{6+} $4f_{7/2}$	35.13	1.65	1388.98
W^{5+} $4f_{5/2}$	36.02	1.65	1834.89
W^{5+} $4f_{7/2}$	33.70	1.65	2446.59

100 mA cm^{-2}

Name	Peak position (eV)	FWHM	Peak area
W^{6+} $4f_{5/2}$	37.27	1.65	1160.88
W^{6+} $4f_{7/2}$	35.13	1.65	1547.89
W^{5+} $4f_{5/2}$	36.02	1.65	1643.94
W^{5+} $4f_{7/2}$	33.70	1.65	2191.95

200 mA cm^{-2}

Name	Peak position (eV)	FWHM	Peak area
W^{6+} $4f_{5/2}$	37.27	1.65	942.19
W^{6+} $4f_{7/2}$	35.13	1.65	1256.24
W^{5+} $4f_{5/2}$	36.02	1.65	1709.11
W^{5+} $4f_{7/2}$	33.70	1.65	2278.86

Supplementary References

1. Wang, J. et al. Grain-boundary-engineered La_2CuO_4 perovskite nanobamboos for efficient CO_2 reduction reaction. *Nano Lett.* **21,** 980-987 (2021).
2. Chen, S. et al. Highly selective carbon dioxide electroreduction on structure-evolved copper perovskite oxide toward methane production. *ACS Catal.* **10,** 4640-4646 (2020).
3. Zhu, J. et al. Cation-deficiency-dependent CO_2 electroreduction over copper-based ruddlesden-popper perovskite oxides. *Angew. Chem. Int. Ed.* **61,** e202111670 (2022).
4. Mignard, D. et al. Revisiting strontium-doped lanthanum cuprate perovskite for the electrochemical reduction of CO_2. *J. CO_2 Util.* **5,** 53-59 (2014).
5. Singh, R. P. et al. Electrochemical insights into layered La_2CuO_4 perovskite: Active ionic copper for selective CO_2 electroreduction at low overpotential. *Electrochim. Acta* **326,** 134952 (2019).
6. Xu, Z. et al. High-rate CO_2-to-CH_4 electrosynthesis by undercoordinated Cu sites in alkaline-earth-metal perovskites with strong basicity. *Adv. Energy Mater.* **13,** 2204417 (2023).
7. Peng, C. et al. Highly-exposed single-interlayered Cu edges enable high-rate CO_2-to-CH_4 electrosynthesis. *Adv. Energy Mater.* **12,** 2200195 (2022).
8. Peng, C. et al. Lithium vacancy-tuned [CuO_4] sites for selective CO_2 electroreduction to C_{2+} products. *Small* **18,** 2106433 (2022).
9. Xiong, L. et al. Geometric modulation of local CO flux in Ag@Cu_2O nanoreactors for steering the CO_2RR pathway toward high-efficacy methane production. *Adv. Mater.* **33,** 2101741 (2021).
10. Tan, X. et al. Stabilizing copper by a reconstruction-resistant atomic Cu-O-Si interface for electrochemical CO_2 reduction. *J. Am. Chem. Soc.* **145,** 8656-8664 (2023).
11. Zhou, X. et al. Stabilizing Cu^{2+} ions by solid solutions to promote CO_2 electroreduction to methane. *J. Am. Chem. Soc.* **144,** 2079-2084 (2022).
12. Peng, C. et al. Lithiation-enabled high-density nitrogen vacancies electrocatalyze CO_2 to C_2 products. *Adv. Mater.* **33,** 2103150 (2021).
13. Wang, Y.-R. et al. Implanting numerous hydrogen-bonding networks in a Cu-porphyrin-based nanosheet to boost CH_4 selectivity in neutral-media CO_2 electroreduction. *Angew.*

Chem. Int. Ed. **60,** 21952-21958 (2021).

14. Li, Y. et al. Promoting CO_2 methanation via ligand-stabilized metal oxide clusters as hydrogen-donating motifs. *Nat. Commun.* **11,** 6190 (2020).
15. Hung, S.-F. et al. A metal-supported single-atom catalytic site enables carbon dioxide hydrogenation. *Nat. Commun.* **13,** 819 (2022).
16. Shi, G. et al. Constructing Cu-C bonds in a graphdiyne-regulated Cu single-atom electrocatalyst for CO_2 reduction to CH_4. *Angew. Chem. Int. Ed.* **61,** e202203569 (2022).
17. Chen, S. et al. Lewis acid site-promoted single-atomic Cu catalyzes electrochemical CO_2 methanation. *Nano Lett.* **21,** 7325-7331 (2021).
18. Hong, S. et al. Tuning the C_1/C_2 selectivity of electrochemical CO_2 reduction on Cu–CeO_2 nanorods by oxidation state control. *Adv. Mater.* **35,** 2208996 (2023).
19. Patra, K. K. et al. Boosting electrochemical CO_2 reduction to methane via tuning oxygen vacancy concentration and surface termination on a copper/ceria catalyst. *ACS Catal.* **12,** 10973-10983 (2022).
20. Wang, R. et al. Partial coordination-perturbed Bi-copper sites for selective electroreduction of CO_2 to hydrocarbons. *Angew. Chem. Int. Ed.* **60,** 19829-19835 (2021).
21. Dai, Y. et al. Manipulating local coordination of copper single atom catalyst enables efficient CO_2-to-CH_4 conversion. *Nat. Commun.* **14,** 3382 (2023).
22. De Gregorio, G. L. et al. Facet-dependent selectivity of Cu catalysts in electrochemical CO_2 reduction at commercially viable current densities. *ACS Catal.* **10,** 4854-4862 (2020).
23. Wang, X. et al. Efficient methane electrosynthesis enabled by tuning local CO_2 availability. *J. Am. Chem. Soc.* **142,** 3525-3531 (2020).
24. Zhao, J. et al. Modulation of $^*CH_xO$ adsorption to facilitate electrocatalytic reduction of CO_2 to CH_4 over Cu-based catalysts. *J. Am. Chem. Soc.* **145,** 6622-6627 (2023).
25. Wang, X. et al. Gold-in-copper at low *CO coverage enables efficient electromethanation of CO_2. *Nat. Commun.* **12,** 3387 (2021).
